# Meta-Analysis of *MMP2*, *MMP3*, and *MMP9* Promoter Polymorphisms and Head and Neck Cancer Risk

**DOI:** 10.1371/journal.pone.0062023

**Published:** 2013-04-24

**Authors:** Caiyun Zhang, Chao Li, Minhui Zhu, Qingzhou Zhang, Zhenghua Xie, Gang Niu, Xicheng Song, Lei Jin, Guojun Li, Hongliang Zheng

**Affiliations:** 1 Department of Otorhinolaryngology-Head and Neck Surgery, Changhai Hospital, Second Military Medical University, Shanghai, China; 2 Department of Head & Neck Surgery, Sichuan Province Cancer Hospital & institute, Chengdu, China; 3 Department of Otolaryngology-Head and Neck Surgery, Yuhuangding Hospital of Qingdao University, Yantai, China; 4 Department of Stomatology, Jinling Hospital, School of Medicine, Southern Medical University, Nanjing, China; 5 Department of Head and Neck Surgery, The University of Texas MD Anderson Cancer Center, Houston, Texas, United States of America; Baylor College of Medicine, United States of America

## Abstract

**Background:**

The 1306 C>T, 1171 5A>6A, and 1562C>T polymorphisms of matrix metalloproteinase (*MMP*) 2, *MMP3,* and *MMP9* genes, respectively, have been found to be functional and may contribute to head and neck carcinogenesis. However, the results of case-control studies examining associations between *MMP* polymorphisms and head and neck cancer (HNC) risk remain inconclusive. Therefore, we performed a meta-analysis to further evaluate the role of these polymorphisms in HNC development.

**Methods:**

We searched PubMed, ISI Web of Knowledge, MEDLINE, Embase, and Google Scholar to identify all published case-control studies of *MMP2*-1306 C>T, *MMP3*-1171 5A>6A, and *MMP9*-1562 C>T polymorphisms and HNC risk in the meta-analysis. Odds ratios (ORs) and 95% confidence intervals (CIs) were used to assess the association between these polymorphisms and HNC risk.

**Results:**

Thirteen studies were included in this meta-analysis. For *MMP2*-1306 C>T polymorphism, significant associations were observed under three genetic models both in overall comparison and in a hospital-based subgroup, and in oral cavity cancer and nasopharyngeal cancer under dominant model as well. For *MMP3*-1171 5A>6A and *MMP9*-1562 C>T polymorphisms, no association was found in overall comparison; however, in subgroup analyses based on ethnicity and tumor site, significant associations were detected between the *MMP3*-1171 5A>6A polymorphism and HNC risk in a European population and pharyngeal/laryngeal cancer under two genetic contrasts.

**Conclusion:**

This meta-analysis suggests that the *MMP2*-1306 C>T polymorphism is associated with HNC risk, as is the *MMP3*-1171 5A>6A polymorphism specifically in some subgroups. Further studies with larger sample sizes are warranted.

## Introduction

Head and neck cancer (HNC), which includes cancers of the oral cavity, pharynx, hypopharynx, and larynx, is one of the most common cancers worldwide [1]. It accounts for nearly 3% of all incident malignancies in the United States with an estimated 52,610 new cases and 11,500 deaths from HNC in 2012 [2]. It is characterized by local tumor aggressiveness that could lead to a high recurrence rate and a low survival rate [3]. Many factors, such as tobacco use, alcohol consumption, viral infection, and genetic susceptibility, are associated with an increased risk of HNC [4]–[6]. Although tobacco smoking and alcohol consumption play a critical role in HNC carcinogenesis, only a small proportion of smokers and drinkers are ultimately diagnosed with HNC. This implies that genetic susceptibility to HNC varies among individuals in the general population [7].

Studies have demonstrated that Matrix metalloproteinases (MMPs) may play an important role in HNC development [8]. MMPs are a family of zinc-dependent proteinases that are capable of degrading essentially all extracelluar matrix components, which is a key event in the invasion and metastasis of most malignancies [9]–[12]. Under normal conditions, MMPs are implicated in both tissue regeneration and wound repair, as well as reproduction [13]–[15]. MMPs may also contribute to carcinogenesis, as previous studies have indicated that MMPs are involved in several steps of cancer development, including cancer cell growth, differentiation, apoptosis, migration, invasion, and metastasis [12].

*MMP2*, *MMP3*, and *MMP9* are three important members of the *MMP* family. *MMP2* (gelatinase- A), located on chromosome 16q13–q21, digests gelatin (denatured collagen), type IV collagen, and some bioactive molecules, such as growth factor-binding proteins and growth factor receptors [16]–[18]. *MMP3* (stromelysin-1), located on chromosome 11q22.2–22.3, can lyse the collagen present in the basal membrane and induces the synthesis of other MMPs such as *MMP1* and *MMP9* [19], [20]. *MMP9* (gelatinase B) is the most complex member of the MMPs family in terms of domain structure. It is capable of degrading decorin, elastin, fibrillin, laminin, gelatin, and types IV, V, XI, and XVI collagen [21], [22]. Overexpression of *MMP2, MMP3,* and *MMP9* has been found to associate with the development of cancer, including HNC [8], thereby indicating that these MMPs may also be implicated in HNC development.

Several polymorphisms in the promoter regions of the *MMP2*, *MMP3*, and *MMP9* genes have been well described. Previous researchers reported that these polymorphisms play critical roles in the regulation of *MMP* gene transcription. *MMP2* -1306 C>T (rs243865), which contains a C to T transition at −1306, is associated with high transcriptional activity of the *MMP2* gene [23]. *MMP3* -1171 5A>6A (rs3025058), which is characterized by the insertion or deletion of a single adenosine at position −1171, could alter *MMP3* transcription levels [24]. *MMP9* -1562 C>T (rs3918242), which includes a C>T transition at position −1562 near the upstream transcription initiation site, also influences the *MMP9* transcriptional levels [25]. Several epidemiologic studies of the association of these three polymorphisms with HNC risk have been carried out [26]–[37]; however, their results remain inconclusive. Thus, we conducted a meta-analysis of all eligible case-control studies published to date to further evaluate the associations between these three polymorphisms and HNC risk.

## Materials and Methods

### Search Strategy

Using key words search in the PubMed, Web of Knowledge, MEDLINE, Embase, and Google Scholar electronic databases and search engines, we identified all eligible case-control studies of the associations of *MMP2, MMP3* and *MMP9* polymorphisms with HNC risk conducted between January 2000 and June 2012. We used the following key words:

“MMP”, “matrix metalloproteinase”, “collagenase”, “gelatinase”, “matrilysin” or “PUMP” and “head and neck cancer”, “oral cancer”, “pharyngeal cancer”, “hypopharyngeal cancer” or “laryngeal cancer” and “polymorphism”, “variant”, “genotype” or “SNP”. After performing the electronic key word searches, we manually reviewed the references of the search results to identify additional evaluable studies. We contacted authors directly for important data that were not reported in original articles. Abstracts, unpublished reports, and articles not written in English were not included.

### Data Extraction

The following details were extracted from each article included in the meta-analysis: first author, publication year, ethnicity of the study population (categorized as Asian and European), the number of cases and controls, and genotype distribution, genotyping methods, allele frequency, and so on. To minimize bias and improve reliability, two investigators extracted the data independently and reached a consensus on all items (the details of each study) via discussion.

### Inclusion and Exclusion Criteria

Studies were included if they: (1) were case-control studies, (2) assessed the associations between *MMP2, MMP3, and MMP9* polymorphisms and HNC risk, (3) had sufficient available data to calculate an odds ratio (OR) with a 95% confidence interval (CI) and P- value, and (4) were published in English.

Studies were excluded if they: (1) had insufficient information about genotype frequency or number, (2) if the same population was evaluated in two or more studies, only the most recent or the one with the largest study population was included in this meta-analysis.

### Statistical Analysis

We evaluated the association of *MMP* polymorphisms and HNC risk using ORs and 95% CIs. The significance of pooled ORs was estimated via a Z test (P<0.05 was considered statistically significant). Heterogeneity between studies was assessed via Cochran’s chi-square Q statistic test. A random- effects model was used when the P value for heterogeneity was less than 0.05, which indicated obvious heterogeneity of the data; otherwise, a fixed-effects model was used. Heterogeneity across studies was also detected using an I^2^ test. As a guide, I^2^ values of <25% were considered low, I^2^ values of 25 to 75% were considered moderate, and I^2^ values of >75% were considered high [38]. The associations between *MMP2, MMP3, and MMP9* polymorphisms and the risk of HNC were evaluated using a recessive genetic model (BB versus AB+AA), dominant genetic model (BB+AB versus AA), and allele contrast model (B-allele versus A-allele), respectively (A represented major allele and B represented minor allele). In addition to overall comparison, subgroup analyses based on the ethnicity of each study population and the source of the control subjects were also performed using different genetic models. Furthermore, sensitivity analyses were performed to reflect the influence of the individual dataset on the pooled ORs by sequential removing each eligible study. Finally, we assessed the publication bias using Begg’s funnel plot and Egger’s test. Additionally, the Hardy–-Weinberg equilibrium (HWE) was calculated via a chi-square test at a significance level of α <0.05. All P values were two-sided, and all statistical analyses were performed using STATA 12.0 software (Stata Corporation, College Station, TX, USA).

## Results

### Study Characteristics

**Figure 1:**
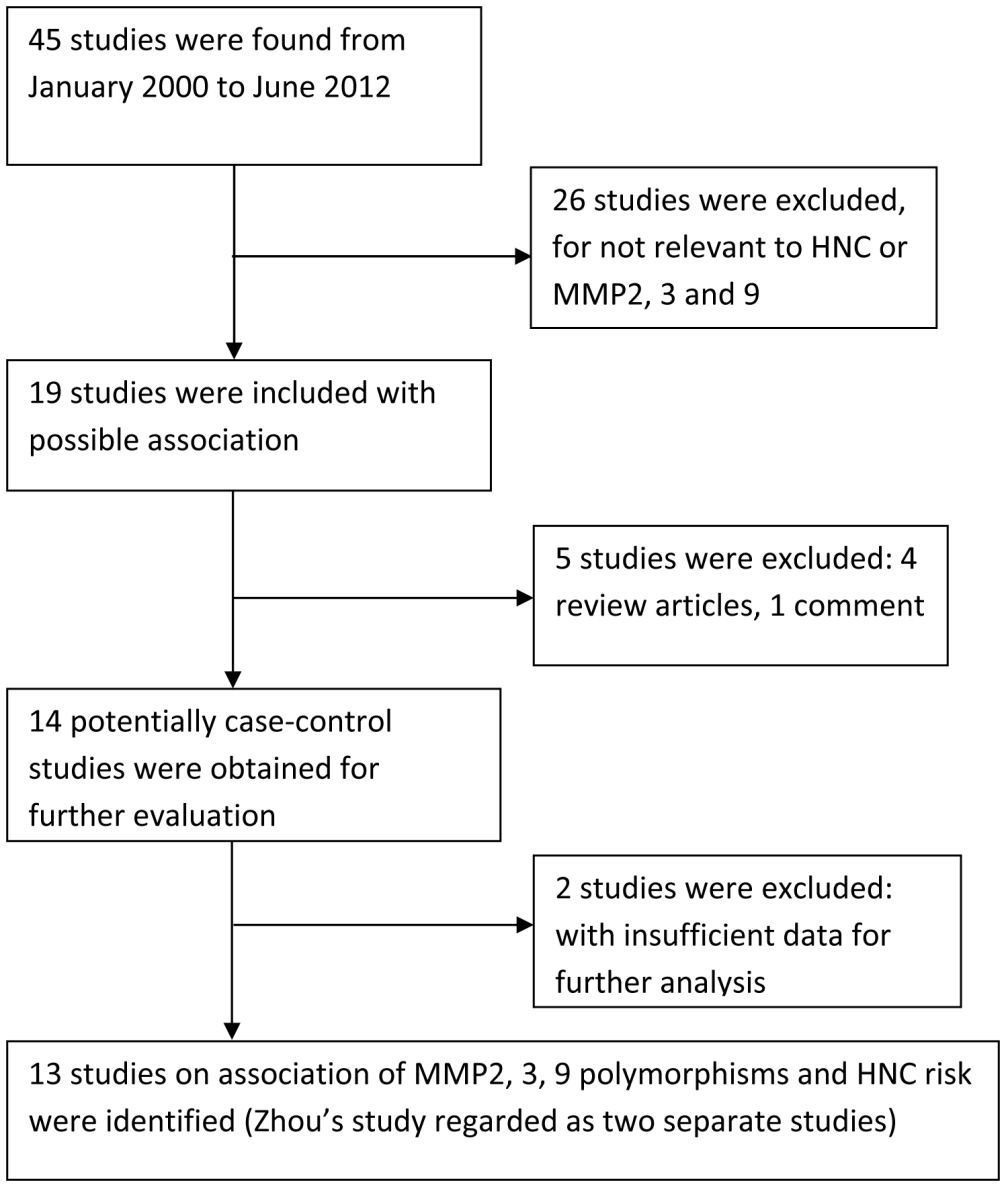
PRISMA flowchart of study identification.

We identified 45 relevant articles using the aforementioned search strategy. However, 33 studies were excluded: 26 did not assess the association between *MMP2*, *MMP3*, and *MMP9* polymorphisms and HNC risk; 2 had insufficient data for further analysis; 4 were review articles; and one was a commentary. Zhou [28] evaluated *MMP2*, *MMP3*, and *MMP9* polymorphisms in a case–-control study of two independent populations. Each population was regarded as a separate study. Consequently, 13 studies of the association of *MMP2*, *MMP3*, and *MMP9* polymorphisms with the risk of HNC were ultimately included in this mata-analysis (Figure 1). Table 1 illustrates the characteristics of all the included studies, such as their publication year, the ethnicity of the study population, tumor site, genotyping data, and sample size (case vs. controls). All the articles included in the meta-analysis were published in English. Polymerase chain reaction–- restriction fragment length polymorphism was the most commonly used genotyping method in these studies. The results from chi-square tests showed that genotypic distribution of the controls was in agreement with the HWE except one study [36] at a statistical significance level of 0.05.

**Table 1:** Characteristics of 13 case-control studies included in this meta-analysis.

Gene	Year	Ethnicity	Tumor site	Source of control	Case				Control				Genotyping method	P_HWE_ 3
					N	AA	AB	BB	N	AA	AB	BB		
**MMP2-1306 C>T**														
Lin	2004	Asian	Oral cavity	HB^1^	121	101	20	0	147	107	34	6	PCR&dHPLC	0.133
O-charoenrat	2006	Asian	Mixed HNC	HB	239	206	33	0	250	190	56	4	PCR-RFLP	0.957
Zhou	2007	Asian	Nasopharynx	PB^2^	570	520	50	0	473	402	70	1	PCR	0.255
Zhou	2007	Asian	Nasopharynx	PB	233	216	17	0	286	244	42	0	PCR	0.18
**MMP3-1171 5A >6A**														
Chaudhary	2010	Asian	Mixed HNC	HB	135	6	23	106	126	2	14	110	PCR-RFLP^4^	0.068
Vairaktaris	2007	European	Oral cavity	HB	160	36	84	40	156	30	75	51	PCR-RFLP	0.796
Hashimoto	2004	Asian	Mixed HNC	HB	140	3	30	107	223	5	63	155	PCR-RFLP	0.634
Nishizawa	2007	Asian	Oral cavity	PB	170	3	50	117	164	8	54	102	PCR-RFLP	0.805
Tu	2006	Asian	Oral cavity	PB	150	0	31	119	98	1	12	85	PCR-RFLP	0.446
Zinzindohoue	2004	European	Mixed HNC	HB	125	36	70	19	249	60	121	68	PCR-RFLP	0.669
Zhou	2007	Asian	Nasopharynx	PB	561	4	103	454	479	3	77	399	PCR	0.731
Zhou	2007	Asian	Nasopharynx	PB	231	4	46	181	284	2	77	205	PCR	0.067
**MMP9-1562 C>T**														
Tu	2007	Asian	Oral cavity	PB	192	144	43	5	191	140	50	1	PCR-RFLP	0.118
Nasr	2007	African	Nasopharynx	PB	174	139	32	3	171	139	31	1	PCR-RFLP	0.604
Zhou	2007	Asian	Nasopharynx	PB	569	454	113	2	480	384	94	2	PCR	0.135
Zhou	2007	Asian	Nasopharynx	PB	234	190	44	0	276	208	67	1	PCR	0.068
Vairaktaris	2008	European	Oral cavity	HB	152	84	68	0	162	114	48	0	PCR-RFLP	0.027

1 HB: hospital-based,

2 PB: population-based,

3 HWE: Hardy-Weinberg equilibrium,

4 PCR-RFLP: Polymerase chain reaction-restriction fragment length polymorphism.

### Quantitative Data Synthesis

**Figure 2:**
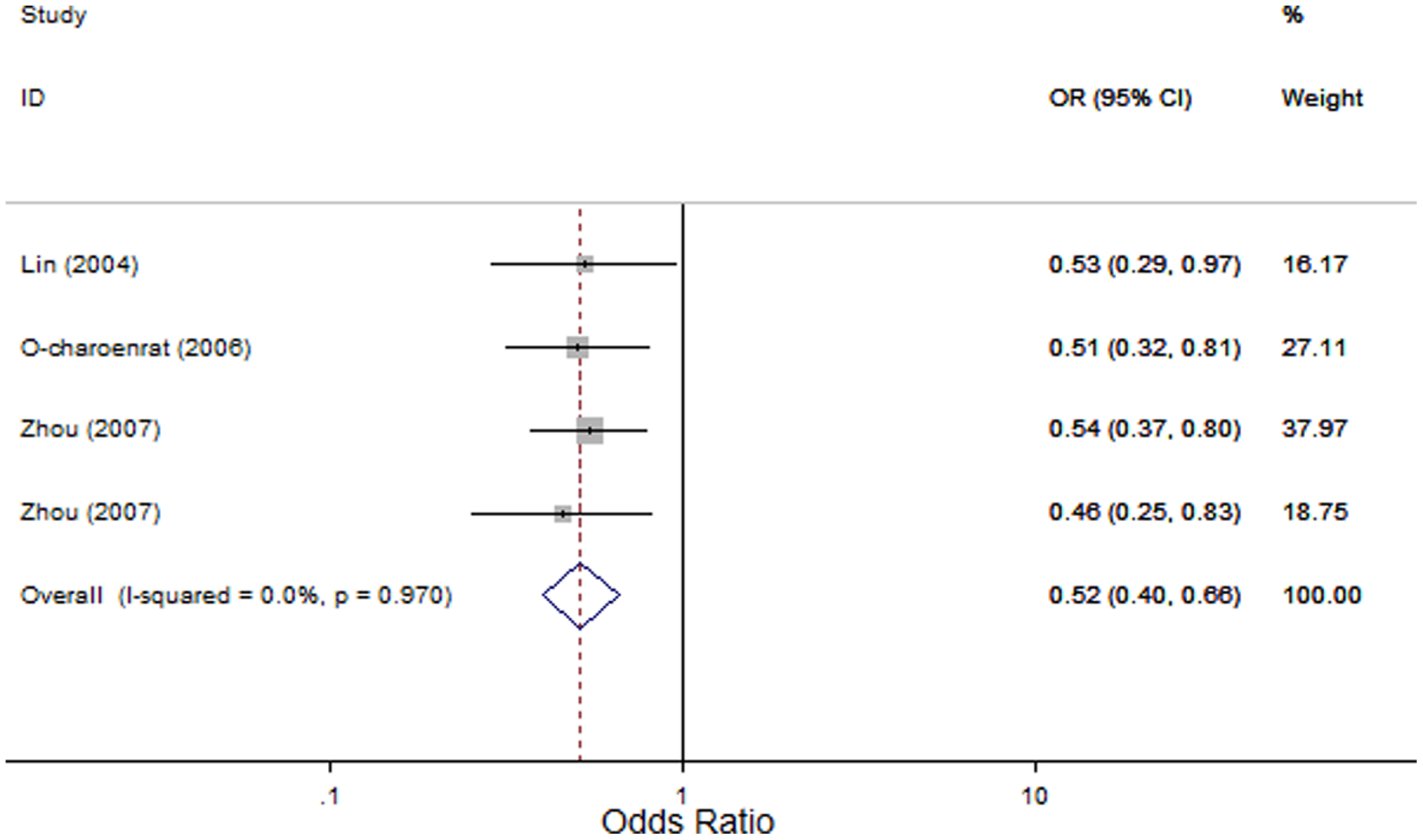
Forest plot for association between *MMP2*-1306 C>T and risk of head and neck cancer under dominant model (CT+TT *vs*. CC). A fixed-effects model was used. The *squares* and *horizontal lines* represent the study-specific OR and 95% CI. The *diamond* corresponds to the summary OR and 95% CI.

*MMP2*-1306 C>T: Four studies evaluated the association of the *MMP2*-1306 C>T polymorphism with HNC risk [26]–[28] with 1163 cases and 1156 controls. In the overall comparison, significant associations between the *MMP2*-1306 C>T polymorphism and HNC risk were observed using three genetic models (OR, 0.12; 95% CI, 0.02–0.69; I^2^, 0, *P_heterogeneity_* = 0.865 for the recessive model; OR, 0.52; 95% CI, 0.40–0.66; I^2^, 0, *P_heterogeneity_* = 0.97 for the dominant model; and OR, 0.52; 95% CI, 0.41–0.65; I^2^, 0, *P_heterogeneity_* = 0.963 for the allele contrast model; Figure 2). Similarly, in subgroup analyses based on the source of control subjects and tumor site, the *MMP2*-1306 C>T polymorphism was significantly associated with HNC risk in the hospital-based subgroup (OR, 0.10; 95% CI, 0.01–0.78; I^2^, 0, *P_heterogeneity_* = 0.907 for the recessive model; OR, 0.52; 95% CI, 0.36–0.75; I^2^, 0, *P_heterogeneity_* = 0.911 for the dominant model; and OR, 0.50; 95% CI, 0.35–0.70; I^2^, 0, *P_heterogeneity_* = 0.913 for the allele contrast model); in the population-based subgroup (OR, 0.52; 95% CI, 0.37–0.71; I^2^, 0, *P_heterogeneity_* = 0.628 for the dominant model and OR, 0.53; 95% CI, 0.39–0.73; I^2^, 0, *P_heterogeneity_* = 0.662 for the allele contrast model); in the oral cavity cancer (OR, 0.47; 95% CI, 0.31–0.73; I^2^, 0, *P_heterogeneity_* = 0.607 for the dominant model); and in the nasopharyngeal cancer (OR, 0.52; 95% CI, 0.37–0.71; I^2^, 0, *P_heterogeneity_* = 0.628 for the dominant model; Table 2).

**Table 2:** Stratified analysis of *MMP2*, *MMP3*, and *MMP9* polymorphisms on HNC risk.

Variables	N^a^	Recessive genetic model (BB *vs*. AB+AA)			Dominant genetic model(BB+AB *vs.* AA)			Allele contrast(B *vs.* A)		
		OR (95% CI)	*P* b	I^2^	OR (95% CI)	*P*	I^2^	OR (95% CI)	*P*	I^2^
**MMP2-1306 C>T**										
Source of control										
Hospital-based	2	0.10(0.01, 0.78)*	0.907	0	0.52(0.36, 0.75)*	0.911	0	0.50(0.35, 0.70)*	0.913	0
Population-based	2	0.28(0.01, 6.80)	N/A	N/A	0.52(0.37, 0.71)*	0.628	0	0.53(0.39, 0.73)*	0.662	0
Tumor site										
Oral cavity	2	N/A	N/A	N/A	0.47(0.31, 0.73)*	0.607	0	N/A	N/A	N/A
Pharynx/larynx	1	N/A	N/A	N/A	0.59(0.34, 1.03))	N/A	N/A	N/A	N/A	N/A
Nasopharynx	2	N/A	N/A	N/A	0.52(0.37, 0.71)*	0.628	0	N/A	N/A	N/A
Total	4	0.12(0.02, 0.69)*	0.865	0	0.52(0.40, 0.66)*	0.97	0	0.52(0.41, 0.65)*	0.963	0
**MMP3-1171 5A>6A**										
Ethnicity										
Asian	6	1.00^c^(0.73, 1.37)	0.026	60.8	1.00(0.54, 1.84)	0.308	16.4	0.99^c^(0.75, 1.31)	0.026	60.6
European	2	0.59(0.41, 0.85)*	0.339	0	0.80(0.56,1.15 )	0.906	0	0.76(0.61, 0.94)*	0.6	0
Source of control										
Hospital-based	4	0.72^c^(0.44, 1.20)	0.016	71.1	0.78(0.55, 1.09)	0.763	0	0.81^c^(0.59, 1.11)	0.048	62
Population-based	4	1.03(0.73, 1.46)	0.064	58.7	1.32(0.61, 2.85)	0.262	24.9	1.04(0.78, 1.39)	0.102	51.7
Tumor site										
Oral cavity	6	0.89(0.62, 1.29)	0.071	50.8	0.94(0.62, 1.42)	0.37	7.3	0.96(0.80, 1.15)	0.067	51.6
Pharynx/larynx	2	0.45(0.28, 0.72)*	0.658	0	0.75(0.46, 1.23)	0.513	0	0.66(0.49, 0.88)*	0.172	46.5
Nasopharynx	2	1.07(0.66, 1.74)	0.061	71.5	0.62(0.20, 1.89)	0.501	0	1.00(0.80, 1.26)	0.122	58.3
Total	8	0.87^c^(0.65, 1.17)	0.004	66.4	0.85(0.62, 1.16)	0.505	0	0.92^c^(0.74, 1.14)	0.013	60.4
**MMP9-1562 C>T**										
HWE	4	1.87(0.66, 5.26)	0.469	0	0.93(0.76, 1.13)	0.527	0	0.96(0.79, 1.15)	0.465	0
Tumor site										
Oral cavity	2	N/A	N/A	N/A	1.33^C^(0.64, 2.74)	0.026	79.9	1.30(0.80, 2.10)	0.099	63.4
Nasopharynx	3	N/A	N/A	N/A	0.93(0.73, 1.18)	0.329	10	0.94(0.74, 1.19)	0.292	18.7
Total	5	1.87(0.66, 5.26)	0.469	0	1.06^c^(0.78, 1.43)	0.037	60.7	1.05(0.89, 1.25)	0.082	51.7

a Number of comparisons.

b P-value for Q-test.

c Random-effects model was used when P-value of Q-test for heterogeneity <0.05, otherwise fixed-effects model was used.

* Statistically significant, with P<0.05.

**Figure 3:**
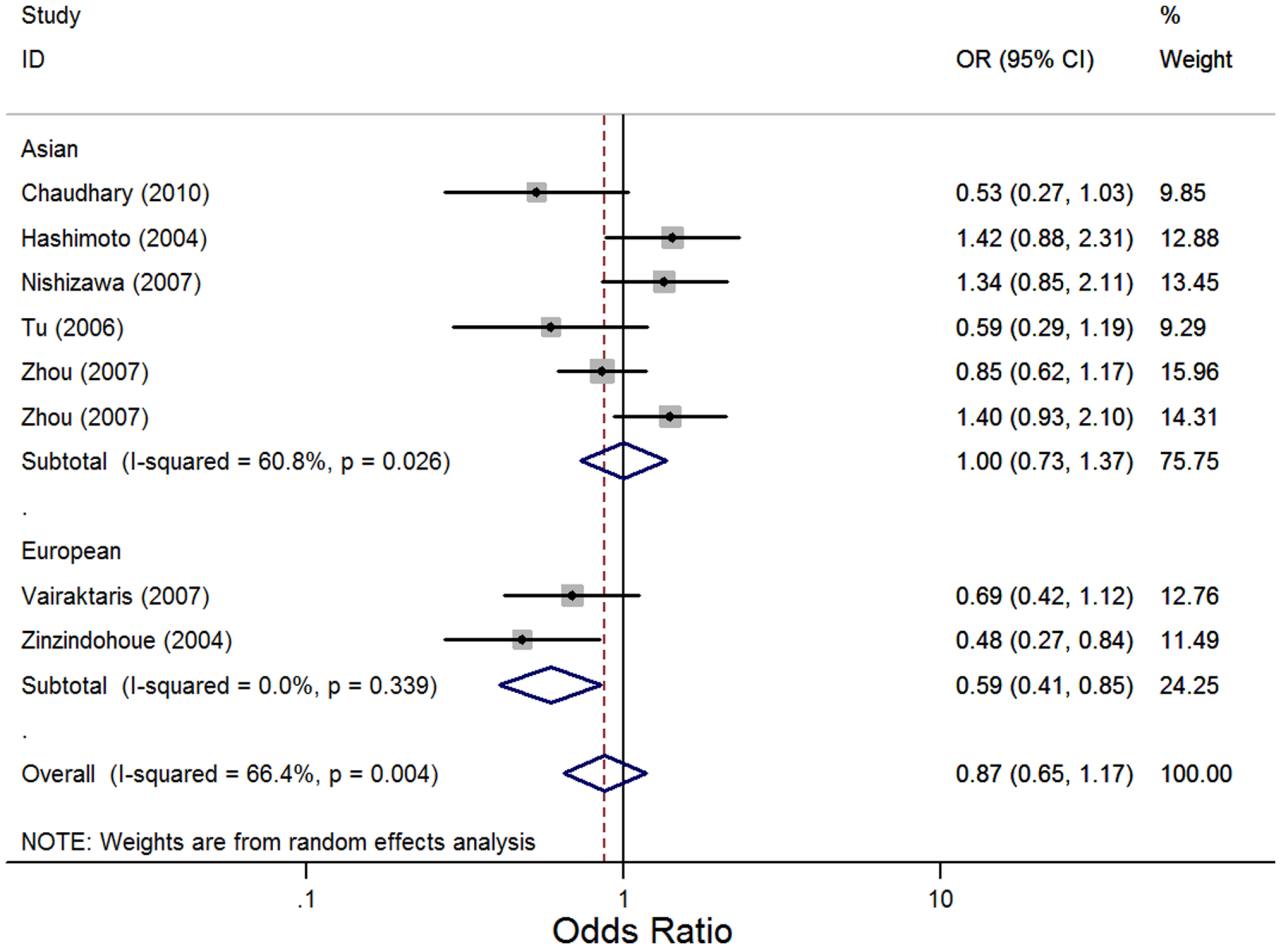
Forest plot for association between *MMP3*-1171 5A>6A and risk of head and neck cancer stratified by ethnicity under recessive model (6A/6A *vs.* 5A/5A+5A/6A). A random effects model was used. The *squares* and *horizontal lines* represent the study-specific OR and 95% CI. The *diamond* corresponds to the summary OR and 95% CI.

*MMP3*-1171 5A>6A: We identified eight studies that evaluated the association of the *MMP3*-1171 5A>6A polymorphism with the risk of HNC [28]–[34] with 1672 cases and 1779 controls. In the overall comparison, the *MMP3*-1171 5A>6A polymorphism was not significantly associated with HNC risk using three different genetic models (OR, 0.87; 95% CI, 0.65–1.17; I^2^, 66.4%, *P_heterogeneity_* = 0.004 for the recessive model; OR, 0.85; 95% CI, 0.62–1.16; I^2^, 0, *P_heterogeneity_* = 0.505 for the dominant model; and OR, 0.92; 95% CI, 0.74–1.14; I^2^, 60.4%, *P_heterogeneity_* = 0.013 for the allele contrast model). However, in subgroup analyses based on ethnicity and tumor site, the *MMP3*-1171 5A>6A polymorphism was significantly associated with HNC risk in Europeans (OR, 0.59; 95% CI, 0.41–0.85; I^2^, 0, *P_heterogeneity_* = 0.339 for the recessive model and OR, 0.76; 95% CI, 0.61–0.94; I^2^, 0, *P_heterogeneity_* = 0.6 for the allele contrast model) and in pharyngeal/laryngeal cancers (OR, 0.45; 95% CI, 0.28–0.72; I^2^, 0, *P_heterogeneity_* = 0.658 for the recessive model and OR, 0.66; 95% CI, 0.49–0.88; I^2^, 46.5%, *P_heterogeneity_* = 0.172 for the allele contrast model), but the *MMP3*-1171 5A>6A polymorphism was not significantly associated with HNC risk in Asians, in oral cavity cancer and in nasopharyngeal cancer using three genetic models (Figure 3). In stratified analyses based on the source of control subjects, the *MMP3*-1171 5A>6A polymorphism was not significantly associated with HNC risk in either the population-based subgroup or the hospital-based subgroup (Table 2).

**Figure 4:**
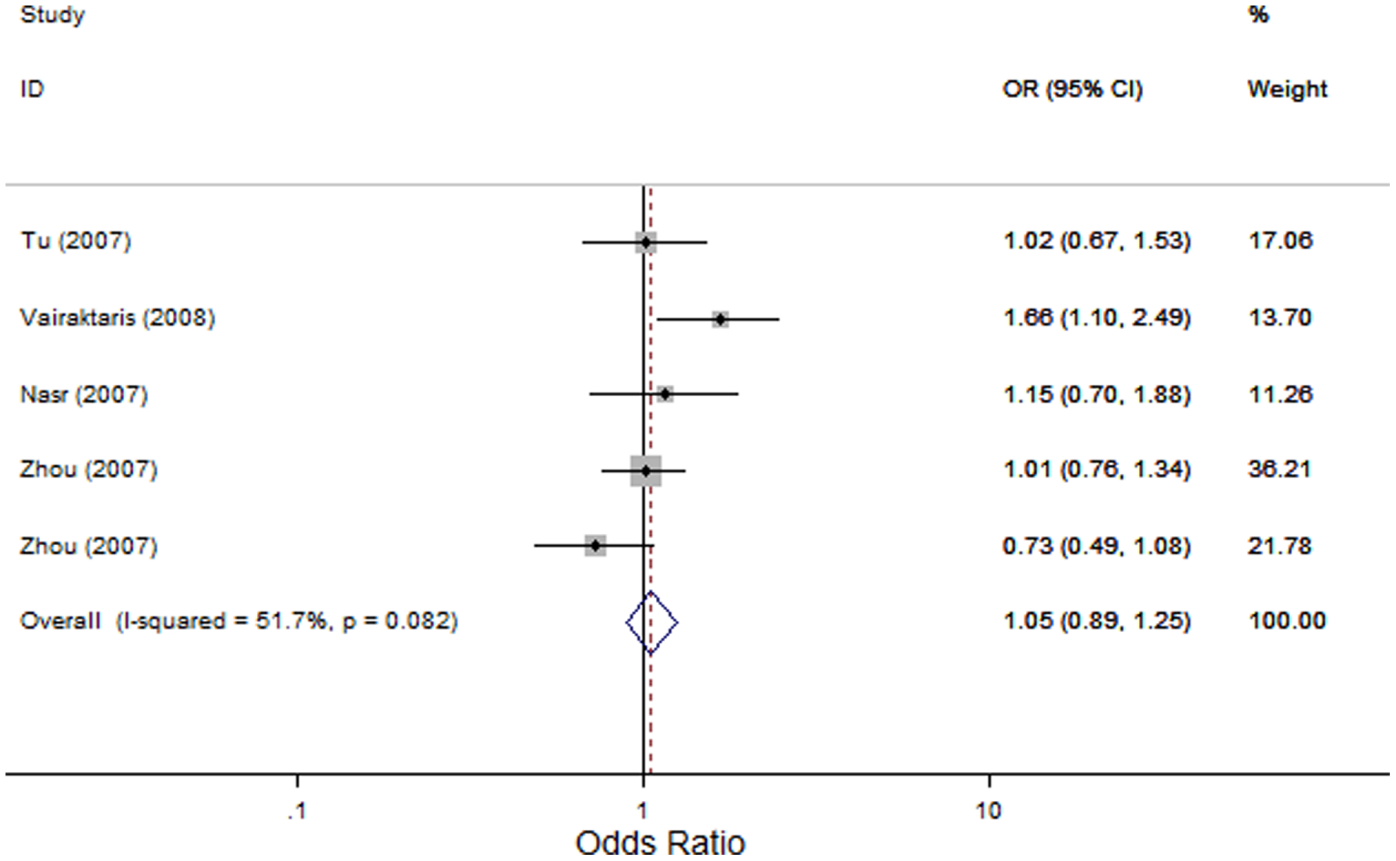
Forest plot for association between *MMP9*-1562 C>T and risk of head and neck cancer under allele contrast (T-allele *vs.* C-allele). A fixed-effects model was used. The *squares* and *horizontal lines* represent the study-specific OR and 95% CI. The *diamond* corresponds to the summary OR and 95% CI.

*MMP9*-1562 C>T: We identified five studies that evaluated the association of the *MMP9*-1562 C>T polymorphism with the risk of HNC [28], [35]–[37] with 1321 cases and 1280 controls. In the overall comparison, the *MMP9*-1562 C>T polymorphism was not significantly associated with HNC risk using three genetic models (OR, 1.87; 95% CI, 0.66–5.26; I^2^, 0 *P_heterogeneity_* = 0.469 for the recessive model; OR, 1.06; 95% CI, 0.78–1.43; I^2^,60.7%, *P_heterogeneity_* = 0.037 for the dominant model; and OR, 1.05; 95% CI, 0.89–1.25; I^2^, 51.7%, *P_heterogeneity_* = 0.082 for the allele contrast model; Figure 4). Similarly, in the subsequent analysis of HWE studies, excluding the study by Vairaktaris and colleagues [35], did not reveal any significant associations between the *MMP9*-1562 C>T polymorphism and HNC risk (OR, 1.87; 95% CI, 0.66–5.26; I^2^, 0 *P_heterogeneity_* = 0.469 for the recessive model; OR, 0.93; 95% CI, 0.76–1.13; I^2^,0, *P_heterogeneity_* = 0.527 for the dominant model; and OR, 0.96; 95% CI, 0.79–1.15; I^2^, 0, *P_heterogeneity_* = 0.465 for the allele contrast model). Furthermore, in subgroup analysis based on tumor site, no significant association was detected either in oral cavity cancer or in nasopharyngeal cancer.

### Heterogeneity Analysis

In specific comparisons, the data from two of the three polymorphisms were hetergeneous. For the *MMP2*-1306 C>T polymorphism, no significant heterogeneity was found either in overall comparison (I^2^ = 0, *P_heterogeneity_* = 0.865 for the recessive model; I^2^ = 0, *P_heterogeneity_* = 0.97 for the dominant model; and I^2^ = 0, *P_heterogeneity_* = 0.963 for the allele contrast model) or in subgroup analyses using three genetic models (Table 2). For the *MMP3*-1171 5A>6A polymorphism, significant heterogeneity was observed in overall comparison using the recessive model (I^2^ = 66.4%, *P_heterogeneity_* = 0.004) and the allele contrast model (I^2^ = 60.4%, *P_heterogeneity_* = 0.013). However, heterogeneity was eliminated in the European population after stratifying by ethnicity (I^2^ = 0, *P_heterogeneity_* = 0.339 for the recessive model and I^2^ = 0, *P_heterogeneity_* = 0.600 for the allele contrast model). Also, in subgroup analyses based on the source of control subjects, heterogeneity significantly decreased in the population-based subgroups (I^2^ = 58.7%, *P_heterogeneity_* = 0.064 for the recessive model and I^2^ = 51.7%, *P_heterogeneity_* = 0.102 for the allele contrast model). For the *MMP9*-1562 C>T, significant heterogeneity was detected using the dominant model. However, when the study by Vairaktaris and colleagues [35], in which genotypic distribution of the controls was not consistent with HWE, was excluded, heterogeneity was not detected, and the significance of pooled ORs using the dominant model was not influenced, thereby suggesting that this study was the major source of heterogeneity.

### Sensitivity Analysis

**Figure 5:**
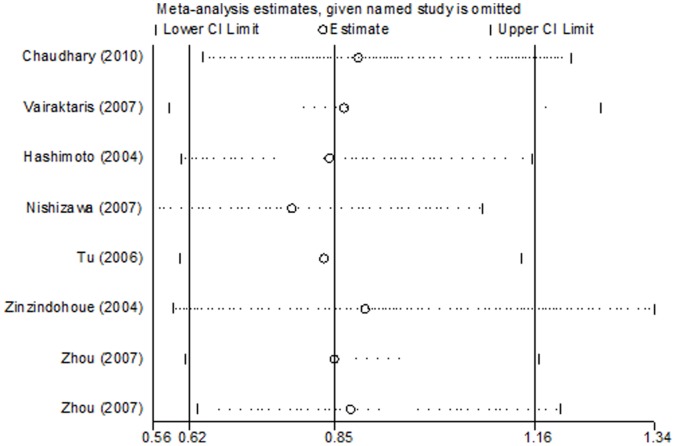
Sensitivity analysis of *MMP3*-1171 5A>6A via the deletion of one study at a time to reflect the influence of the individual dataset on the pooled ORs using dominant model.

Sensitivity analyses were performed to assess the influence of individual dataset on the pooled ORs by sequential removing each eligible study. For *MMP2*-1306 C>T, the results demonstrated that the significance of pooled ORs was undetectable after excluding the studies [26], [27] from a recessive model (data not shown). For *MMP3*-1171 5A>6A and *MMP9*-1562 C>T, the significance of the pooled ORs was not materially altered by exclusion of any individual study (Figure 5), thereby indicating that our results are statistically robust.

### Publication Bias

**Figure 6:**
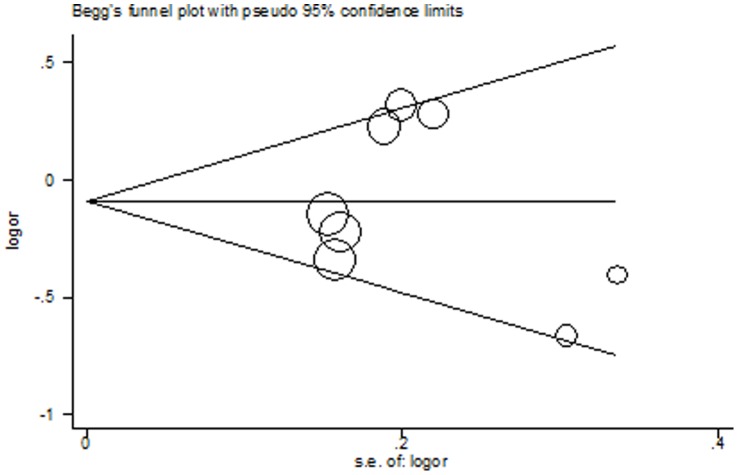
Begg’s funnel plot of *MMP3*-1171 5A>6A for publication bias test. Each point represents a separate study for the indicated association under allele contrast.

For all the three polymorphisms, the shapes of the Begg’s funnel plots in all genetic models did not show any evidence of obvious asymmetry. Figure 6 shows the shape of the Begg’s funnel plots of *MMP3*-1171 5A>6A using allele contrast model. Moreover, Egger’s test did not reveal any significant evidence of publication bias of all the three polymorphisms (data not shown).

## Discussion

In this meta-analysis, the *MMP2*-1306 C>T polymorphism was significantly associated with HNC risk both in overall comparison and in subgroup analyses based on the source of the controls and tumor sites. In contrast, no association was observed between either *MMP3*-1171 5A>6A or *MMP9*-1562 C>T polymorphism and HNC risk in overall comparison; however, in subgroup analyses based on ethnicity and tumor site, significant associations were found between the *MMP3*-1171 5A>6A polymorphism and HNC risk in Europeans and pharyngeal/laryngeal cancer under two genetic contrasts. Our findings indicate that *MMP2*-1306 C>T polymorphism might modulate risk of HNC, so does the *MMP3*-1171 5A>6A polymorphism in some subgroups.

The *MMP2*-1306 C>T polymorphism, which contains a C>T transition at the −1306 position upstream of the transcriptional site, can abolish Sp1-binding site and downregulate transcriptional activity. Previous studies have shown that *MMP2* gene expression was significantly lower in individuals with the T allele than in individuals with the C allele [23]. Our meta-analysis indicates that individuals with variant genotypes (CT or TT genotype) are less susceptible to HNC than individuals with the wild genotype (CC genotype). However, our findings confirmed those of previous studies [8], [23] which reported that *MMP2* overexpression was associated with the development and aggressiveness of a variety of malignancies including HNC, as most patients in the studies included in our meta-analysis carried the C allele but not the T allele.

For example, O-Charoenrat and colleagues assessed the association of the *MMP2*-1306 C>T polymorphism and its expression level with the risk of HNC [27]. They found that the C and T allele frequencies were 93.1% and 6.9%, respectively, in patients, compared with 87.2% and 12.8%, respectively, in controls (P<0.05), and the CC genotype frequencies were significantly higher in patients than in controls (86.2% *vs*. 76%; P<0.05). Moreover, they also found that *MMP2* expression in HNC cells containing the CC genotype was significantly higher than that in cells with the CT genotype. Similarly, in a study of the association of the *MMP2*-1306 C>T polymorphism with the risk of oral squamous cell carcinoma (OSCC) [26], Lin and colleagues reported that the CC genotype frequency was significantly higher in OSCC cases than in controls (P = 0.04). However, because of the small samples and limited number of studies, our results should be interpreted with caution. Further studies with larger samples are needed to validate our findings.

For the *MMP3*-1171 5A>6A polymorphism, functional analysis *in vitro* showed that the 5A allele had approximately 2-fold higher promoter activity than the 6A allele. This finding implies that the 5A allele is responsible for increased *MMP3* transcriptional levels and contributes to the carcinogenesis of most malignancies. Several groups have evaluated the association between *MMP3*-1171 5A>6A polymorphism and the risk of HNC; however, the results of these studies remain inconsistent. Chaudhary and colleagues found that the 5A allele might play an important role in the susceptibility to HNC, as individuals with 5A/5A genotype had nearly two fold risk of HNC (OR = 1.94) when compared to controls [29]. However, Tu and colleagues found that the 5A/5A genotype was associated with the risk of oral submucous fibrosis but not OSCC [33]. Similarly, in studies by Nishizawa and Hashimoto, no significant association between the *MMP3*-1171 5A>6A polymorphism and HNC risk was found, which is consistent with the findings of this meta-analysis [31], [32]. However, in our meta-analysis, the *MMP3*-1171 5A>6A polymorphism was significantly associated with risk of HNC in Europeans when the study population was stratified by ethnicity, thereby indicating that the discrepancies in the aforementioned results may be attributed to diverse genetic backgrounds and different environmental factors in different populations. Future studies with larger samples are warranted to further evaluate the role of the *MMP3*-1171 5A>6A polymorphism in HNC risk in different populations.

The *MMP9*-1562 C>T polymorphism, which is located at position 1562 bp upstream of the transcriptional start site and contains either C or T, has been shown to influence the transcriptional activity of the *MMP9* gene. Zhang and colleagues performed transient transfection and DNA-protein interaction assays and found that T allele-associated promoter activity was higher than the C allele-associated promoter activity owing to the binding of a transcriptional repressor [25]. Although *MMP9* plays an important role in head and neck carcinogenesis and *MMP9* is frequently overexpressed in HNC, our meta-analysis indicated no significant association between the *MMP9*-1562 C>T polymorphism and HNC risk, suggesting that *MMP9* expression might influence HNC progression via mechanisms other than regulation by the *MMP9*-1562 C>T polymorphism. Several other factors, such as interleukin-1, tumor necrosis factor α, and oncogenes, may also regulate *MMP9* expression [21], [39]. Further studies are needed to test these hypotheses.

Some heterogeneity factors between studies that could limit the strengths of the meta-analysis should be addressed. First, ethnicity was one of the most important factors that could lead to heterogeneity because of the diverse genetic backgrounds and environmental factors in different ethnicities. Second, tumor site was another reason for the heterogeneity between studies as HNC have quite different origins of organs, different histological subtypes, different etiology and different biological behavior. For example, tobacco use and alcohol consumption play important roles in oral cavity cancer, while viral infection is the major risk factor for oropharyngeal and nasopharyngeal cancer. Thus, different risk factors for different tumor sites may explain why the same polymorphism may play different roles in different subgroups of HNC. Furthermore, the source of the controls was another factor that could lead to heterogeneity. Population-based controls could be more reliable than hospital-based controls because the genotype distributions in hospital-based controls may be deviated from normal. Thus, population-based study design for individual subgroups of HNC is needed for future studies.

Since this is a pooled analysis, we thus have had relatively higher study power for the evaluation of such associations. In addition, we have performed stratified analysis by tumor sites in this meta-analysis, while our analysis by different tumor sites might minimize the issue of the confounding effect from mixed tumor sites. Although this analysis had such strengths, it also had some limitations. First, the number of eligible studies included in this meta-analysis was limited, and the sample size of each study was relatively small, especially in stratified analyses. For example, there were only two studies examined the association between the *MMP3*-1171 5A>6A polymorphism and HNC risk in Europeans. Although significant association was detected, the statistical power could have been limited. Second, if more detailed information about age, sex, alcohol consumption, tobacco smoking and/or HPV status had been available in the original studies, a more accurate OR would have been estimated after further stratification. Third, evaluating the association between *MMP* polymorphisms and HNC risk using linkage disequilibrium (LD) would have been more powerful. However, few studies performed haplotype analysis of these three *MMPs*. Additionally, publication bias may have occurred because we included only published studies in the meta-analysis, although it was not detected via a statistical test. Despite these limitations, however, the statistical power of the analysis could have been significantly increased as the cases and controls were pooled from different studies. Therefore, our results from this meta-analysis might be more reliable than those of individual studies.

In conclusion, this meta-analysis suggests that the *MMP2*-1306 C>T polymorphism is associated with the risk of HNC, as is the *MMP3*-1171 5A>6A polymorphism specifically in some subgroups. However, the *MMP9*-1562 C>T polymorphism is not associated with HNC risk. Further studies with larger samples are warranted to further evaluate the association between *MMP* polymorphisms and HNC risk.

## Supporting Information

Table S1 **PRISMA 2009 Checklist.**
(PDF)

## References

[1] Jemal A, Bray F, Center MM, Ferlay J, Ward E, et al. (2011) Global cancer statistics. CA Cancer J Clin 61: 69–90. 21296855 10.3322/caac.20107

[2] Siegel R, Naishadham D, Jemal A (2012) Cancer Statistics. CA Cancer J Clin 62: 10–29. 22237781 10.3322/caac.20138

[3] Vokes EE, Weichselbaum RR, Lippman SM, Hong WK (1993) Head and neck cancer. N Engl J Med 328: 184–194. 8417385 10.1056/NEJM199301213280306

[4] Sankaranarayanan R, Nair MK, Mathe WB, Balaram P, Sebastian P, et al (1992) Recent results of oral cancer research in Kerala, India. Head Neck 14: 107–112. 1601646 10.1002/hed.2880140206

[5] Kabat GC, Chang CJ, Wynder EL (1994) The role of tobacco,alcohol use, and body mass index in oral and pharyngeal cancer. Int J Epidemiol 23: 1137–1144. 7721514 10.1093/ije/23.6.1137

[6] Blot WJ, McLaughlin JK, Winn DM, Austin DF, Greenberg RS, et al (1998) Smoking and drinking in relation to oral and pharyngeal cancer. Cancer Res 48: 3282–3287. 3365707

[7] Sturgis EM, Wei Q (2002) Genetic susceptibility–molecular epidemiology of head and neck cancer. Curr Opin Oncol 14: 310–317. 11981277 10.1097/00001622-200205000-00010

[8] Stokes A, Joutsa J, Ala-Aho R, Pitchers M, Pennington CJ, et al (2010) Expression profiles and clinical correlations of degradome components in the tumor microenvironment of head and neck squamous cell carcinoma. Clin Cancer Res16: 2022–2035. 10.1158/1078-0432.CCR-09-2525 20305301

[9] Freije JM, Balbin M, Pendas AM, Sanchez LM, Puente XS, et al (2003) Matrix metalloproteinases and tumor progression. Adv Exp Med Biol 532: 91–107. 12908552 10.1007/978-1-4615-0081-0_9

[10] Stetler-Stevenson WG, Yu AE (2001) Proteases in invasion: matrix metalloproteinases. Semin Cancer Biol 11: 143–52. 11322833 10.1006/scbi.2000.0365

[11] Nagase H, Woessner JF (1999) Matrix metalloproteinases. J Biol Chem 274: 21491–21494. 10419448 10.1074/jbc.274.31.21491

[12] Stetler-Stevenson WG, Liotta LA, Kleiner DE (1993) Extra cellular matrix 6: role of matrix metalloproteinases in tumor invasion and metastasis. Faseb J 7: 1434–1441. 8262328 10.1096/fasebj.7.15.8262328

[13] Bellayr IH, Mu X, Li Y (2009) Biochemical insights into the role of matrix metalloproteinases in regeneration: challenges and recent developments. Future Med Chem 1: 1095–1111. 20161478 10.4155/fmc.09.83 PMC2794138

[14] Zhang H, Chang M, Hansen CN, Basso DM, Noble-Haeusslein LJ (2011) Role of matrix metalloproteinases and therapeutic benefits of their inhibition in spinal cord injury Neurotherapeutics. 8: 206–220. 10.1007/s13311-011-0038-0 PMC3077748 21455784

[15] VanSaun MN, Matrisian LM (2006) Matrix metalloproteinases and cellular motility in development and disease. Birth Defects Res C Embryo Today 78: 69–79. 16622849 10.1002/bdrc.20061

[16] Murphy G, Ward R, Hembry RM, Reynolds JJ, Kuhn K, et al (1989) Characterization of gelatinase from pig polymorphonuclear leucocytes. A metalloproteinase resembling tumour type IV collagenase. Biochem J 258: 463–472. 2539808 10.1042/bj2580463 PMC1138384

[17] Woessner JF (1991) Matrix metalloproteinases and their inhibitors in connective tissue remode-ling. Faseb J 5: 2145–2154. 1850705

[18] Nelson AR, Fingleton B, Rothenberg ML, Matrisian LM (2000) Matrix metalloproteinases: biologic activity and clinical implications. J Clin Oncol 18: 1135–1149. 10694567 10.1200/JCO.2000.18.5.1135

[19] Brinckerhoff CE, Rutter JL, Benbow U (2000) Interstitial collagenases as marker of tumor progression. Clin Cancer Res 6: 4823–4830. 11156241

[20] Van Themsche C, Potworowski EF, ST-Pierre Y (2004) Stromelysin-1 (MMP-3) is inducible in T lymphoma cells and accelerates the growth of lymphoid tumors in vivo. Biochem Biophys Res Commun 315: 884–891. 14985095 10.1016/j.bbrc.2004.01.144

[21] Westermarck J, Kahari VM (1999) Regulation of matrix metalloproteinase expression in tumour invasion. FASE 13: 781–792. 10224222

[22] Murphy G, Docherty AJ (1992) The matrix metalloproteinases and their inhibitors. Am J Respir Cell Mol Biol 7: 120–125. 1497900 10.1165/ajrcmb/7.2.120

[23] Price SJ, Greaves DR, Watkins H (2001) Identification of novel, functional genetic variants in the human matrix metalloproteinase-2 gene: role of Sp1 in allele-specific transcriptional regulation. J Biol Chem 276: 7549–7558. 11114309 10.1074/jbc.M010242200

[24] Ye S (2000) Polymorphism in matrix metalloproteinase gene promoters: implication in regulation of gene expression and susceptibility of various diseases. Matrix Biol 19: 623–639. 11102751 10.1016/s0945-053x(00)00102-5

[25] Zhang B, Henney A, Eriksson P, Hamsten A, Watkins H, et al (1999) Genetic variation at the matrix metalloproteinase-9 locus on chromosome 20q12.2–13.1. Hum Genet 105: 418–423. 10598806 10.1007/s004390051124

[26] Lin SC, Lo SS, Liu CJ, Chung MY, Huang JW, et al (2004) Functional genotype in matrix metalloproteinases-2 promoter is a risk factor for oral carcinogenesis. J Oral Pathol Med 33: 405–409. 15250832 10.1111/j.1600-0714.2004.00231.x

[27] O-Charoenrat P, Khantapura P (2006) The role of genetic polymorphisms in the promoters of the matrix metalloproteinase-2 and tissue inhibitor of metalloproteinase-2 genes in head and neck cancer. Oral Oncol 42: 257–267. 16275157 10.1016/j.oraloncology.2005.07.008

[28] Zhou G, Zhai Y, Cui Y, Qiu W, Yang H, et al (2007) Functional polymorphisms and haplotypes in the promoter of the MMP2 gene are associated with risk of nasopharyngeal carcinoma. Hum Mutat 28: 1091–1097. 17607721 10.1002/humu.20570

[29] Chaudhary AK, Singh M, Bharti AC, Singh M, Shukla S, et al (2010) Synergistic effect of stromelysin-1 (matrix metalloproteinase-3) promoter (-1171 5A->6A) polymorphism in oral submucous fibrosis and head and neck lesions. BMC Cancer 10: 369. 20630073 10.1186/1471-2407-10-369 PMC2912870

[30] Vairaktaris E, Yapijakis C, Vasiliou S, Derka S, Nkenke E, et al (2007) Association of -1171 promoter polymorphism of matrix metalloproteinase-3 with increased risk for oral cancer. Anticancer Res 27: 4095–4100. 18225577

[31] Hashimoto T, Uchida K, Okayama N, Imate Y, Suehiro Y, et al (2004) Association of matrix metalloproteinase (MMP)-1 promoter polymorphism with head and neck squamous cell carcinoma. Cancer Lett 211: 19–24. 15194213 10.1016/j.canlet.2004.01.032

[32] Nishizawa R, Nagata M, Noman AA, Kitamura N, Fujita H, et al (2007) The 2G allele of promoter region of matrix metalloproteinase-1 as an essential pre-condition for the early onset of oral squamous cell carcinoma. BMC Cancer 7: 187. 17919326 10.1186/1471-2407-7-187 PMC2089080

[33] Tu HF, Liu CJ, Chang CS, Lui MT, Kao SY, et al (2006) The functional (-1171 5A–>6A) polymorphisms of matrix metalloproteinase 3 gene as a risk factor for oral submucous fibrosis among male areca users. J Oral Pathol Med 35: 99–103. 16430740 10.1111/j.1600-0714.2006.00370.x

[34] Zinzindohoué F, Blons H, Hans S, Loriot MA, Houllier AM, et al (2004) Single nucleotide polymorphisms in MMP1 and MMP3 gene promoters as risk factor in head and neck squamous cell carcinoma. Anticancer Res 24: 2021–2026. 15274394

[35] Vairaktaris E, Vassiliou S, Nkenke E, Serefoglou Z, Derka S, et al (2008) A metalloproteinase-9 polymorphism which affects its expression is associated with increased risk for oral squamous cell carcinoma. Eur J Surg Oncol 34 450–455. 17498910 10.1016/j.ejso.2007.03.024

[36] Nasr HB, Mestiri S, Chahed K, Bouaouina N, Gabbouj S, et al (2007) Matrix metalloproteinase-1 (-1607) 1G/2G and -9 (-1562) C/T promoter polymorphisms: susceptibility and prognostic implications in nasopharyngeal carcinomas. Clin Chim Acta 384: 57–63. 17599818 10.1016/j.cca.2007.05.018

[37] Tu HF, Wu CH, Kao SY, Liu CJ, Liu TY, et al (2007) Functional -1562 C-to-T polymorphism in matrix metalloproteinase-9 (MMP-9) promoter is associated with the risk for oral squamous cell carcinoma in younger male areca users. J Oral Pathol Med 36: 409–414. 17617834 10.1111/j.1600-0714.2007.00552.x

[38] Higgins JPT, Thompson SG, Deeks JJ, Altman DG (2003) Measuring inconsistency in meta-analyses. Br Med J 327: 557–560. 12958120 10.1136/bmj.327.7414.557 PMC192859

[39] Yoshizaki T, Sato H, Furukawa M, Pagano JS (1998) The expression of matrix metalloproteinase 9 is enhanced by Epstein–Barr virus latent membrane protein 1. Proc Natl Acad Sci 95: 3621–3626. 9520415 10.1073/pnas.95.7.3621 PMC19885

